# Systems thinking and modelling to support transformative change: key lessons from inter-disciplinary analysis of socio-ecological systems in applied land systems research

**DOI:** 10.1007/s43621-025-00987-3

**Published:** 2025-04-01

**Authors:** Miriam Glendell, Matt Hare, Kerry A. Waylen, Kerr Adams, Jean Léon Boucher, Zisis Gagkas, Alessandro Gimona, Simone Martino, Keith B. Matthews, J. Gareth Polhill

**Affiliations:** 1 Environmental and Biochemical Sciences Department, The James Hutton Institute, Craigiebuckler, Aberdeen, AB15 8QH UK; 2 Information and Computational Sciences Department, The James Hutton Institute, Craigiebuckler, Aberdeen, AB15 8QH UK; 3 The James Hutton Institute, Social, Economic and Geographical Sciences Department, Craigiebuckler, Aberdeen, AB15 8QH UK

**Keywords:** Systems thinking, System modelling, Sustainable development goals, Global change, System transformation

## Abstract

The evolving ‘permacrisis’ of compounding environmental and social challenges calls for transformative approaches to understanding and intervening in socio-ecological systems. Approaches to support systems thinking and understanding can be vital to achieving this goal. However, applying such systems thinking is often challenging, and we need to better reflect on the pros and cons of different approaches for building systems understanding and informing changes. In this paper, we first identify key attributes of systems thinking approaches from literature. We then use these as a framework for comparing and evaluating seven different systems thinking approaches, selected on the basis of our experience in applying them in support of the management and governance of various types of land systems. The seven approaches are: agent-based modelling, Bayesian belief networks, causal loop modelling, spatial multicriteria analysis, societal metabolic analyses, social network mapping and quantitative story telling. This framework has allowed us to appraise and reflect on our own experiences to identify the respective strengths and weaknesses of these different methodologies. We note that some of the ability to inform change depends as much on the context within which specific tools are used as the particular features of the tools themselves. Based on our appraisal, we conclude by suggesting six key recommendations that should be followed by others seeking to commission and use systems approaches, in order to enable them to support transformative change. We hope this may be useful to those working with systems approaches, since there is an urgent need for analytic efforts that can inform and enable transformative change. We also reiterate the call for sustained funding for long-term, standards-based evaluation of systems thinking approaches with respect to whether their use can demonstrate instrumental impacts leading to the kind of transformation the IPCC has called for, i.e. fundamental system change that goes beyond capacity development impacts such as network-building.

## The challenge

Despite the large body of evidence that human activities threaten natural systems and hence our own wellbeing, researchers are frustrated by the slow pace of action in the face of the urgent challenges presented by global environmental crises [1]. The impacts of these crises, including climate change, biodiversity loss, pollution, and resource depletion, are widely recognised as critical, interconnected threats to society and human wellbeing. Over the past two decades, these emergencies have become part of the mainstream discourse, reflected in the United Nations 17 Sustainable Development Goals (SDGs) [2]. Policies and goals that are ostensibly aimed at tackling these crises are being adopted across the world, such as the European Union’s Green Deal [3]. However, implementation of these policies largely falls short of ambitions, as highlighted by the 2023 UN SDG Summit [2]. Progress is slow in tackling climate change [4, 5], even though in many countries it is an issue of widespread societal concern [6], while biodiversity continues to decline at a rate of the ‘Sixth Mass Extinction’ [7].

Better efforts to address these challenges are urgently needed. Recent analysis shows that seven out of eight globally quantified safe and just Earth System Boundaries for climate, biosphere, water and nutrient cycles, and aerosols at global and sub global scales have already been exceeded [8, 9]. The accelerating loss of biodiversity is now, on average, 35 times the “background” rate for many taxa [10], depletion of essential natural resources, such as inorganic phosphate fertilisers [11, 12], as well as socio-economic changes and geopolitical shocks, are contributing to ‘global systemic risks’ [13]. Should these drivers coalesce, they may lead to a ‘global polycrisis’ with irreversible consequences for humanity [13].

Many of these challenges are interlinked, as recognised by analysis of the efforts needed to meet the SDGs [14] as well as the parallel development of academic concepts such as *wicked problems* [15] and the *water, energy, and food nexus* [16]. However, policies still predominantly reflect sectoral objectives in so-called ‘silos’, without considering complex linkages, interdependencies, and feedbacks. Thus, more integrated long-term policies [17] and interventions are needed, informed by knowledge, understanding and approaches that support *systems thinking* [18, 19] (see definition in Sect. 2). Public policies are a major driver of both socio-ecological challenges and responses; hence informing, altering, or transforming policymaking is a key target for activities and tools designed to promote change informed by systems thinking. In fact, systems thinking is beginning to be institutionally endorsed by some governments, including the UK Department of Environment, Food and Rural Affairs [20] and the European Commission [17]. In the academic world, this is reflected in a recent drive towards funding of integrated inter-disciplinary science by major research organisations (e.g. [21]). A systemic ‘challenge-driven’ approach to research and innovation [17] is called for to understand the causes that might lead to an irreversible ‘polycrisis’ and generate actionable policies to mitigate this risk [13].

When addressing the pressing imperatives of the climate and biodiversity crises, systems thinking is called upon to incorporate this complexity, reconcile multiple land use objectives and identify key levers and leverage points for transformative action. Landscapes are not only physical spaces where many of these challenges converge, but also offer an opportunity to consider how socio-economics interacts with the implementation of integrated environmental policies in the context of biophysical processes and constraints. Industrial agriculture has been successful in feeding people, but at the cost of simplifying complex social-ecological systems in ways that make them more vulnerable to climate change [7] and geopolitical shocks [17]. Concentrating land use on a single ecosystem service compromises benefits from others, such as greenhouse gas mitigation, freshwater quality [22] and biodiversity restoration [23, 24]. The need for a holistic approach to transforming the food system was recognised at COP28 as a vital part of global climate change mitigation and adaptation activities [25]. We believe systems thinking approaches provide a tool for dealing with this level of complex, interacting challenges. Therefore, in this Perspective piece, we provide a set of examples assessing systems thinking approaches in the context of applied land systems research, defined as “terrestrial social-ecological systems where human and environmental systems interact through land use” [26].

In Sect. 2 we first define what we know and understand under ‘systems thinking’ and review its key attributes as described in the literature. In Sect. 3, we apply these attributes as a framework to support the comparison and multi-criteria evaluation of seven systems thinking approaches that we have direct experience of using in land systems research. Comparisons between the approaches are made according to our experiences of their strengths and weaknesses as well as their ability to support key attributes of systems thinking and their ability to A) represent a system; B) foster systems thinking; and C) support transformative change. Based on this, in Sect. 4 we discuss lessons from the integration of these systems thinking approaches into decision-making and the ability of such analysis to affect real-world transformative change.

## What is systems thinking and what are its key attributes?

At a rudimentary level, a system can be defined as “a regularly interacting or interdependent group of items forming a unified whole” [27]. Some systems are more complex than others and none exist independently of other systems. It is important, in analysis, to define their extents and boundaries, and relations to other systems, especially those they may be embedded in. Systems also take inputs from and send outputs to other systems. As a result, systems can be something of a ‘black box’ to human perception, and analysts must define how we will study and think about them. As defined, some systems may include human activities, and some may not. Moreover, different systems will require different treatments if any type of transformation is desired. To ‘think’ like systems, ‘systems thinkers’ will need to properly define, circumscribe, and understand their systems.

Our starting point for identifying key characteristics of systems thinking is the definition provided by Arnold and Wade [28]. Based on their wide-ranging review of systems thinking, they propose the following:

“Systems thinking is a set of synergistic analytic skills used to improve the capability of identifying and understanding systems, predicting their behaviours, and devising modifications to them in order to produce desired effects. These skills work together as a system.” (p. 675).

In Table 1, we present key attributes of systems thinking that support the goals contained in the above definition. To build this list, we use Arnold and Wade’s [28] requirements for systems thinking approaches, and add to these other attributes identifiable in seminal works, such as Meadow’s *Thinking in Systems* [29] and Schelling’s work on complexity and emergence [30], as well as making use of more recent primers [20, 29, 30]. Finally, this list is informed by the literature on knowledge exchange for decision-making on the environment [31, 32]. This results in 26 attributes, which can be used to appraise and compare a diversity of tools or approaches intended to support systems thinking. We then decided to group these attributes according to their ability to: A) represent a system; B) foster systems thinking; and C) support transformative change (Table 1). The first two “abilities” we consider as fundamental to the successful implementation of systems approaches, whilst the third is fundamental to supporting the area of application related to the topic of our paper: transformation.

**Table 1:** Key attributes of systems thinking identified from various sources [20], [28–30] and by the authors. These have been ordered according to the source and then grouped by ability to: A) represent a system; B) foster systems thinking; and C) support transformative change

	Key attributes of systems thinking	Group	Source
1	Recognizing interconnections within the system, i.e. permitting the identification of the myriad ways in which system components interact with each other	A	[28]
2	Identifying and understanding feedback loops, i.e. the positive and negative feedback cycles that occur in systems	A	
3	Understanding system structure through Abstraction, i.e. the ability to explain the complexity of a system, its components and causal chains and feedback loops through generalisations	A	
4	Identifying dynamic behaviour, i.e. how the system changes over time under different contexts	A	
5	Explaining dynamic behaviour, i.e. what makes the system change over time	A	
6	Differentiating types of stocks, flows, variables, i.e. different elementary parts of the system	A	
7	Identifying and understanding non-linear relationships, i.e., interactions between system components that do not result in consistent uni-directional change	A	
8	Reducing complexity by modelling systems conceptually and visually, i.e. the ability to explain complex systems through modelling in a way that makes them simple to understand	C	
9	Understanding embedded systems at different scales, i.e., the ability to consider or represent within larger systems descriptions, the sub-systems that occur, as well as the latter’s subsystems, and so on	A	
10	Predict – “foretell as a deducible consequence”, i.e. to identify how the system will probably change in response to an event, for example	C	
11	Integrate diverse opinion, i.e. to be able to represent or consider the full range of perspectives based on lived experience or scientific knowledge, for example	B/C	[20]
12	Represent and or manage uncertainty, i.e. to be able to express and deal with aspects of the system that are little known	B/C	
13	Analyse and manage instability of the system, i.e., to understand and then reduce unwanted fluctuations in the system	B/C	
14	Identify long-term solutions, i.e. solutions that will work for a given, long period of time	C	
15	Locate and understand how to correctly push leverage points, i.e. the key “places to intervene in the system” (p. 145) for maximum impact	C	[29]
16	The ability to identify key states and thresholds of the system beyond which the system might transform very rapidly with or without intervention	A	[30]
17	Produces a formal representation of the system as a whole, e.g. a formal logical description, a piece of computer code, or other representational language, such as a directed graph	A/B	
18	Identifies emergent system behaviours, i.e., identify behaviours that are not described in the formal representation of the system components; behaviours that arise from the interaction of the system components	B	
19	Takes into account the heterogeneity of systems elements (e.g. those that impact individuals in different ways to the average impact on the population)	A	
20	Can represent temporal dynamics, i.e. include in its formal representation how and why the system might change over time	A	
21	Devise and test modifications to the system	B/C	
22	Include a wide range of stakeholders in [specifying] system conceptualisation, e.g. through participatory systems approaches	A/B/C	
23	Visualise the system to key stakeholders, i.e., stakeholders that typically have some expert knowledge or lived experience of the system	C	
24	Communicate and visualise the system to wider public, stakeholders that typically will not have lived or expert knowledge of the system	C	
25	Identify systems that are changing structure, i.e. systems that are undergoing changes in the components and interactions that drive them	A	
26	Has proven capability to integrate with other systems approaches, i.e. has been demonstrated to work successfully in combination with other specific systems approaches	C	

## Implementing systems thinking approaches

In this section, we evaluate seven different approaches for supporting systems thinking, i.e.: agent-based modelling (ABM), Bayesian belief networks (BBN), causal loop modelling (CLM), spatial multicriteria analysis (sMCA), societal metabolic analyses (SMA), social network mapping (SNM) and quantitative story telling (QST). This selection is based on the approaches we as authors have had many years of experience in using as part of our research supporting policy for land systems [26]. Consequently, this selection is neither intended to represent a complete set of available approaches, nor for identifying the best ones to use in all circumstances. We believe that sharing our collective experience adds value to potentially more comprehensive evaluations grounded in literature review, which may however lack insights from first-hand application in land systems research. Based on our experience, we first detail, in Table 2, what we consider to be the approaches’ key strengths and weaknesses, as well as their capability to impact on policy and practice beyond academia. In Table 3, by contrast, we then present our multi-criteria evaluation of these approaches, by scoring their ability (none, weak, moderate, strong) to support each of the systems thinking attributes identified in Table 1. Whilst to the best of our knowledge, we are not aware of other research characterising system modelling approaches in a more robust way than we have done, this evaluation – based on our working experience of using the approaches, rather than on theoretical considerations or by literature review–can be considered as a starting point for further discussion using more quantitative approaches. The seven approaches that we evaluate vary greatly in, for example, their disciplinary and epistemological basis and the time and skills required to use them. The ensuing discussion in this section explores their merits and differences further.

**Table 2:** Examples of authors’ applications of seven different systems thinking approaches, along with their strengths, weaknesses and scientific impacts that we have identified. We define impact as ‘an effect on, change or benefit to the economy, society, culture, public policy or services, health, the environment or quality of life, beyond academia’ [33] and base the evaluation on our research experience

Systems thinking approach	Application	Strength	Weaknesses	Impact (low/medium/high) and why?
Spatial multicriteria analysis (sMCA)–explicitly evaluates multiple and potentially conflicting spatial (mapped) criteria to highlight interactions between spatial variables affecting environmental and human systems [34]	Landscape to national level; landscape planning [35], ecosystem services [36]; Digital soil mapping [37, 38] integrated with earth observation [39]	Captures and analyses spatial relationships. Visual communication of results, potentially receiving instant feed-back from stakeholders in successive iterations	Needs to be coupled with other tools for a real system understanding; complexity; data requirements; computational intensity; (sometimes). Need to deal with autocorrelation. Tendency to be overconfident regarding the validity/correctness of maps	High–Indispensable in environmental and urban planning. Government incentives for riparian woodlands informed by results from RIVERTOOL, a spatial tool for multi-criteria analysis of ecosystem services enhancement. https://storymaps.arcgis.com/stories/f49964c9d7344ac4a6056cbde3122946
Bayesian belief network (BBN)–graphical probabilistic causal model of the system [40]	Local to national scale; water quality [41, 42], ecosystem services [43, 44] future scenarios [45] and adaptive catchment management [45]	Strong visual representation allows model co-creation with stakeholders, account for uncertainty. Modular, easy to update parameterisation, allow to test different conceptual understanding by altering model structures. Can be used as a meta-model, integrating insights and strengths from other approaches [46, 47]	Weak on representation of temporal dynamics and feedback loops	Medium – contributed to regulatory assessments. Policy changes in regulatory agency, financial constraints, short-term planning horizons and lack of collaborative governance framework affected implementation of findings [48]
Agent based modelling (ABM)–computer simulation that explicitly represents the interactions of heterogeneous individuals [49]	Local [50] to global [51] scale; wide range of disciplines [52]; past [53] to future [54]	High levels of ontological realism, ease with which knowledge can be integrated from diverse sources [55], spatially-explicit [56], temporally dynamic, low requirement for unrealistic assumptions needed for the sake of simplicity and/or analytical tractability [57]	High data demands for empirical applications, with commensurate issues with access, confidentiality, etc. High computing demands. Interdisciplinary skills needed for ABM rare. Lacks methodologies [49] and code reuse, and there are issues with traditional model quality metrics being inappropriate for complex systems [58]. Prediction in complex systems using ABMs is a long-standing issue of contention [59]^a^	Medium – though potential for ABM to contribute to transformations / transitions has been identified [60], it has yet to gain traction in the area in comparison with more ‘established’ methods [61]. However, there is a considerable and long-standing body of work on using ABM with stakeholders [62–64] including work on visualization [65]
Societal Metabolic Analyses (SMA)–such as Multi-Scale Integrated Analysis of Societal and Ecosystem Metabolism (MuSIASEM) [66]	Local to global, micro to macro analyses of socioecological systems, their interactions and complexity. Can be a city, state, region and tracking of energy and materials use	Strong for robustly demonstrating relational analyses in complex socioecological systems. Like other analyses, much is dependent on availability and quality of data	Data quality and availability challenges, and sometimes a challenge to communicate. Potentially weaker on representing some dynamics and feedbacks	It can be high, but this is up to the actors and decision makers – having and communicating the results of a metabolic analysis is itself not enough for transformation to occur
Social network mapping (SNM)–an approach to build a network of social (actors) and ecological elements (ecosystem services) by deliberation [67, 68]	Social network structure has gained popularity in several fields such as biology [69], physics [70], and public health, with frequent applications to environmental resources and ecosystems management and climate change. SNM has been used to study the human-nature relationships in the context of fisheries [71], wildfires [72], wetlands [73], and watershed governance [74]. At landscape scale has been used for addressing the socio-ecological system of farming in developed [67] and developing countries [67, 68]	The approach provides a democratic way to discuss connections between actors emphasising strong and weak links. Links from multiple actors of the supply chain can be depicted building on a plurality of voices. It can be used to make a qualitative prediction of expected changes that different connections between farms, markets and institutions have on land use and the capacity of the environment to protect natural capital and generate ecosystem services [75]	The network, to reflect a real picture of the social links, needs to consider multiple perspectives. Network mapping can be partial if some stakeholders are not part of the workshops and not reflected by those who take part in it. Some difficulties to link land management and effects on natural capital and ecosystem services may be experienced by the actors participating in the network. Thus, it is good to look for secondary data to fill gaps. Multiple iterations of the network structures are necessary over time to depict temporal change of the social structure and effects on governance and management of natural resources	Medium–this approach may identify bottlenecks, inefficiencies, and pain points within the existing systems, setting the stage for improvement. The few examples of networks linking social and natural capital, mainly targeting the farming context, showed the ability to discover the food-biodiversity nexus [76] and create a more explicit bottom-up collaborative governance framework
Causal loop modelling (CLM)–participatory group model building approach [77], abstract conceptual systems model co-constructed with individuals or groups as a directed cyclic graph	Usable with a wide range of stakeholders from peasant communities to multinational policy makers, scientists to non-experts. Versions in which written model nodes are replaced by icons make it usable by illiterate communities. Applications include water resources management [78]; community-based climate change adaptation planning [79]; rapid response policy modelling [80]; participatory identification of potential impacts of the rise of exascale computing (very fast computers that can operate at 10^18^ floating point operations per second) on the future of agent-based social simulation and the social sciences [81]; and food systems transformation [82]	Can identify system structure, including embedded subsystems, visualise and explain causal system components interconnections and their feedbacks. Structured method for representing and integrating diverse perspectives, allowing the elicitation of tacit knowledge. Can be reasoned about logically to understand dynamic system behaviour and used as the basis of qualitative simulation, or analysed mathematically since the model is equivalent to a matrix. Limited predictive deduction through a basic form of qualitative simulation [83] is possible and it can identify leverage points well	No quantitative computational simulation capabilities and cannot be used to identify threshold effects or tipping points because it cannot properly model temporal behaviour, although limited forms of temporal change can be represented if the syntax and semantics of the model’s edges are altered. Emergent behaviour is not detectable, limited capacity to deal with uncertainty. The directed cyclic graph representation embodies less expressive power than other formal approaches such as systems dynamics modelling or ABM	Low–it can have high social learning and conceptual impacts. However, the possibility of impacting on decision making is dependent on having strong buy-in of decision makers into accepting the outcome of processes. This lack of evidenced impact on action (due in part to not enough commitment to evaluation) is a common problem of participatory modelling of all types [84]
Quantitative story telling (QST)–is a process of using computer-based tools (the quantification) within policy and other decision-making processes with the outputs being narratives (the story telling) that explain both the issues and the agreed responses. This process emphasises agreeing with stakeholders the problem framing, formal representation, choice of performance metrics and their interpretation [85]	Applications have typically been in science for policy analysis at a range of scales [86]	Recognises the importance of the semantic and interpretivist context within which modelling occurs, building on insights from the use of decision support systems. Links with concepts elaborated by modellers themselves [87] and seeks to increase impact (conceptual or instrumental change) by increasing salience and credibility of analysis [88]	Increases the range of skills needed within a modelling team and the resources (time particularly) needed to build relationships. May be epistemically challenging as brings together qualitative and quantitative methods and data. Can emphasise good enough answers rather than innovation	High–formalised processes of science-policy engagement that have been used with policy partners in Government since 2008. Over that period there have been significant conceptual and instrumental changes, influenced by building the salience and credibility of the research outputs using QST [89]. Significant redistributions of agricultural support payments have been underpinned by the analysis and ongoing work continues to support delivery of more transformative change

^a^This statement may seem at odds with the assertion in Table 3 that ABM has ‘strong’ predictive capacity according to *our* evaluation, but what we refer to here is our expert knowledge of contention in the field about prediction, e.g., although Chattoe-Brown [130] makes a compelling case that ABM has valuable roles to play in social science prediction, there are arguments that ABMs cannot be used for prediction, such as those of Edmonds [131]

**Table 3:**
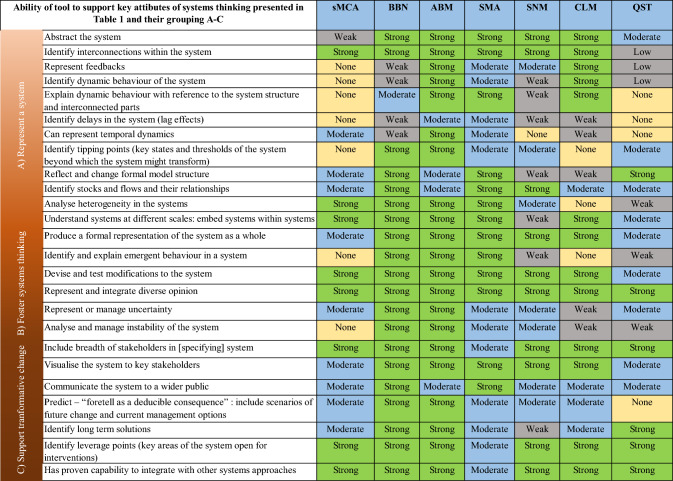
The ability of the seven different systems thinking approaches, we have applied, to support the different attributes of systems thinking shown in Table 1 and thus their capacity to: A) represent the system; B) foster systems thinking; and C) support transformative change. We use a gradient of ability from ‘None’ through ‘Weak’ and ‘Moderate’ to ‘Strong’. Approaches: Spatial Multicriteria Analysis (sMCA); ABM – Agent-based modelling; BBN – Bayesian belief network; SMA – Societal metabolic analyses; SNM – Social Network Mapping; CLM – Causal loop modelling; QST – Quantitative story telling

### A) Representing the system

- *What level of formalism is used by the different approaches?* There are categorical divisions between the modelling approaches we employ in terms of formal (sMCA, BBN, ABM, SMA), partially formal (CLM) and informal (QST). Formal representations are based on formal languages, with agreed rules of inference that have been proven (logically/mathematically) to be internally consistent (i.e. do not lead to the inference that A and not-A are simultaneously true in the same place). Formal languages allow machine processing. Partially formal representations such as those used in CLM employ a formal language (in the case of CLM, this is defined by a syntax of directed graphs combined with the semantics of edge causality and proportionality) which permits a significant amount of rule-guided causal inferencing to be carried out by hand but has not been subjected to a rigorous (i.e. logical) process of consistency checking. Informal representations could range from artistic expression (where there are no universal rules of inference at all) to a ‘partial’ informality in which there are possibly context-sensitive and/or culturally-influenced, underlying ‘rules’ of inference (e.g. ‘common sense’), but they have not been subjected to a rigorous (i.e. logical) process of consistency checking (e.g. QST) and the representation is not underpinned by a defined formal language.^1^ The ability to provide an abstraction of the system is, however, common to all systems modelling approaches considered, with the exception of geospatial analysis.
- There are differences in the *representation of interconnections, feedbacks, delays, temporal dynamics, threshold effects and tipping points.* All approaches, except QST, can identify interconnections within the system. SMA is particularly strong for robustly demonstrating relational analyses in complex socioecological systems, for example agricultural interactions with economy and society [85], where this conceptual framework aided the co-development of system understanding with stakeholders, despite generating uncomfortable knowledge. However, SMA is weaker on quantifying dynamics and feedback. The highest ability to identify feedbacks is supported in ABM and CLM. For SMA, feedbacks are primarily identified in diagnostic mode; for example, the circular way biomass is used in agroecological cycles to regenerate that biomass itself [90]. Identifying system delays is challenging for all system modelling approaches applied in our research. ABM is well suited to representing temporal dynamics while sMCA is strong at identifying trade-offs, and BBN and ABM are strong at identifying threshold effects and tipping points. For example, a coupled agent-based and meta-community model could identify a threshold effect in a non-linear relationship between agri-environmental incentives and biodiversity, when a sharp increase in environmental benefit occurred for a small increase in incentive [91]. Bayesian Networks allow for the investigation of threshold effects and tipping points using discretised variables states, as demonstrated in Adams et al*.* [92], who investigated the impact of climatic, socioeconomic and management scenarios on the probability of surface waters meeting water quality standards. SMA can identify threshold effects and tipping points in simulation. SNM and QST have a weaker ability to represent this factor, while CLM cannot.
- *Understanding dynamic system behaviour and systems that are changing structure.* Only a few approaches can identify and explain dynamic system behaviour (ABM, CLM) [93, 94]. SMA can also explain dynamic behaviour with reference to the system structure and interconnected parts, however this requires in-depth analysis, as in ABM. BBNs, SMA and QST are well suited to reflect and modify system structure, for example when transferring BBN models between different river basins [95].
- *Identifying stocks, flows and heterogeneity in the system.* BBNs, SMA and SNM are suited for the identification of stocks and flows. Heterogeneity in the system can be well represented in sMCA, BBN, ABM and SMA, for example in spatial applications of BBNs to phosphorus and pesticide pollution in river basins [41, 42].
- *Understanding systems at different scales: embedding systems within systems* is possible using most modelling approaches, except for SNM and to a medium effect in QST. For example, CLM is well suited to represent embedded subsystems, visualise and explain causal system components interconnections and their feedback, as demonstrated in e.g. operations research [77]. However, modelling complex processes across scales is often hindered by gaps in process understanding and data pertaining to different scales of interest.

### B) Foster systems thinking

- *Can the formal model representation be explained and changed?* Key to promoting systems thinking is the ability to produce a formal representation of the system that represents diverse opinion and allows testing modifications to the system. This can be accomplished using all approaches reviewed here, although QST has a medium rather than high ability to devise and test modifications to the system. All approaches are capable of representing diverse opinions. For example, CLM has been used with stakeholders from peasant and urban communities [96] to national policy makers [97], to support municipal climate change adaptation planning, and basin-scale flood and drought risk management, respectively. Its pictorial version can even be accessible to illiterate communities. SNM also provides a democratic way to discuss connections between actors, e.g. by quantifying links from multiple actors based on a plurality of voices, provided all key stakeholder voices are represented [98].
- *Does the model represent uncertainty?* Only GIS-supported sMCA, BBN and ABM are strong on representing or managing uncertainty and analysing instability. For example, BBNs can capture uncertainty both in knowledge and data when modelling river basin resilience to future change [45]. Conversely, CLM has limited ability to deal with uncertainty. BBN, ABM and SMA are also capable of identifying and explaining emergent behaviour in a system, which is not possible using CLM.

### C) Supporting transformative change

- *How inclusive can the approach be?* Including breadth of stakeholder views in specifying system conceptualisation is key to all three aspects of model evaluation to represent a system, foster systems thinking and support transformative change. Different modelling approaches are able to include the participation of stakeholders, with BBNs [45], CLM and QST being particularly strong in this respect. For example, QST has been used widely in formalised processes of science-policy engagement with policy partners [99] to inform development of policies that could lead to significant redistribution of agricultural support payments. BBNs are tools that are more regularly used participatively with experts since assessing probabilities is cognitively challenging, for example when understanding the risk of pollutant transfer within river basins [100]. ABM, in their companion modelling format [101] that includes participatory modelling with stakeholders, are a very inclusive approach. Land use applications are wide-ranging, from small-scale farming systems, to forestry, and river basin management [102–104]. Other versions of participatory modelling with ABM permit intermediate levels of inclusion (e.g. [105]). CLM and SNM are extremely inclusive. SMA questions ‘grammars’ – the assumed narratives that stakeholders articulate – and after identifying systemic trade-offs seeks input from stakeholders as to a most desirable way forward. For instance, SMA has been used with QST to engage EU policy stakeholders in how to improve the sustainability performance of agricultural production systems [85]. This is an important part of social learning and promotes systems thinking by building capacities in society. Additionally, in another example, stopping groundwater degradation by implementing nature-based solutions requires effective collaboration among different decision-agents, which can be informed by mapping the complex web of interactions using SNM and simulating different interventions [106].
- *The ability to visualise the system to key stakeholders and communicate it to the wider public* is a key attribute of an effective system analysis approach. Most system approaches applied in our work support this attribute, with some approaches being inherently graphical, such as BBN, and hence more easily communicated, while others require additional custom-visualisation, such as an interactive web-based multi-criteria analysis tool to inform spatial planning of riparian woodlands.^2^ While all modelling approaches require carefully designed bespoke strategies to communicate the system structure and the modelling outcomes to the general public, sMCA, BBNs, SMA, CLM are better suited to communicating system complexities, as they are inherently more visual than ABM, SNM and QST.
- *Ability to predict and foretell, identify leverage points and long-term solutions* are critical attributes to facilitate the usefulness of system thinking outcomes, promote long-term perspectives and enhance resilience. BBNs are well suited to predict or ‘foretell’ deducible consequences, for example the impact of different stakeholder preferences on sustainable catchment management [92]. They can be easily adapted to include scenarios of future change as well as current management options and can even be extended to include counter-factual reasoning [40]. For example, May et al*.* [107] simulated the impacts of future land use change on phosphorus losses to Scottish standing waters using a BBN model and ABM derived CRAFTY-GB land use scenarios [54]. BBNs, ABM, QST are strong at identifying long-term solutions e.g. to increase resilience and efficiency of food supply chains [108]. Multiple system analysis tools are suitable to identify leverage points, including BBN, ABM, SMA, SNM, CLM and QST. For instance, SNM network can help to understand which stakeholders are more relevant to changing natural resource governance to foster transformative change [109].
- *Complementarity of approaches* can be seen and explained by the way different approaches cover different requirements, helping to overcome the limitations of individual tools. Most approaches applied in our work are capable of integration, however sMCA often relies on the results of other tools for a real system understanding, for example coupling with BBN to inform spatial planning in forested landscapes, while considering trade-offs in ecosystem services [50]. BBN can be used as meta-models, integrating results from other modelling approaches, such as process-based models and climate projections to understand future catchment resilience to phosphorus pollution [45] and future pesticide pollution risk in agricultural landscapes [46, 110]. SNM and ABM are also complementary. While ABM simulates network dynamics, SNM focusses on interaction. This allows to identify most suitable networking interventions to overcome collaboration barriers, as in Giordano et al*.* [106] who applied a hybrid SNM-ABM approach to prevent degradation of groundwater status and associated ecosystem services through nature-based solutions. SMA generally covers multiple vertical scales while horizontally integrating different socioecological measures. SMA and QST can be integrated [85]. CLM has been traditionally used to specify system dynamics models [77] and have been successfully used to support the specification of ABM. For example, in [80], participatory causal loop modelling was used to identify the requirements for adapting a legacy ABM – which simulates future changes in the size of cattle farms in Scotland–in order to simulate the potential transformational impacts on farm sizes due to Brexit, as well as the war in Ukraine.

This categorisation and evaluation of the individual system thinking approaches, according to key characteristics they possess, shows that no single approach combines all characteristics and advantages of systems thinking. Best modelling practice now requires multiple models to account for structural model uncertainty and to combine the strengths of different approaches. For example, leveraging SNM’s ability to identify stakeholders critical to influencing change towards effective governance structures, could enhance the ability of other approaches to transfer findings for real-world change [109]. In all cases, accessing pertinent data remains difficult and can only be overcome through consistent implementation of FAIR open science principles [111]. A plurality of approaches and disciplines is therefore needed to build a holistic inter-disciplinary system understanding for sustainability. This need for integrating complementary approaches has also been highlighted by other researchers [112]. Furthermore, there is generally a need for more attention to be given to using systems thinking to achieve change and this requires attention to context and engagements within which analyses may be co-produced, as well as modelled. An effective application of system approaches therefore requires strong interdisciplinary teams, spanning biophysical, computational and social sciences.

## Integrating systems thinking approaches into decision-making and policy for transformative change

If analyses based on systems thinking are to achieve influence, they must be salient, credible and legitimate to those who can make decisions, such as policymakers [88]. This relates to decision-makers’ perceptions of the analysts themselves, datasets as well as the processes in which they are involved. For example, a recent survey of Members of Scottish Parliament (MSPs) found that their most valuable source of information comes from trusted organisations whom they are in regular contact with (Newsdirect, pers. comm. 23. March 2023). This reflects similar, earlier, findings related to the information use hierarchy among water managers [113]. Thus, the context and processes within which specific tools or models are used, and the attributes and efforts of those working with them, are at least as important as the attributes of the model itself. As a result, activities such as building personal connections will assist with knowledge exchange [114].

Systems thinking is an opportunity to empower stakeholders in the decision-making process and co-develop acceptable sustainability pathways [19]. Adopting methods that empower and involve stakeholders throughout the research process, in line with the principles of trans disciplinarity [115] and co-production [116], should offer the best opportunity for systems research to support transformative change [117]. However, such investment in transdisciplinary collaborative work to foster salience, credibility and legitimacy requires effort and capacity from both scientists and policymakers and goes beyond those needed for ‘mere’ systems modelling and analyses [118]. The fundamental complexity of linking participatory systems approaches to decision making perhaps is reflected in the finding that despite significant investment and even best practice [92], this co-creation does not always lead to transformative action. This may be related to a lack of institutional capacity for long-term planning [17] to enable institutions to go beyond the status quo. Overcoming this may go beyond what individual systems modelling projects can achieve, requiring long-term involvement in influencing and perhaps transforming governance structures that can respond to systems analyses, and support integrated systemic approaches. We illustrate this in a case study in Box 1 where a river basin provides a biophysical framework for system-based understanding of interconnected challenges and co-creation of solutions with relevant stakeholders, via the application of BBN. This suggests that considering how to foster collaborative action is as important as endorsing systems models or concepts [119].

Case study (Box 1). Systems thinking in the river Eden basin, Scotland, using BBN. Freshwaters are critical for supporting both the natural environment and human health [120]. In a river basin system, freshwater is shared across human systems required for multiple ecosystem services. In the river Eden basin, Scotland, interactions between human systems and changing environmental conditions are leading to compounding water quality, flooding and drought issues. The river Eden flows through prime agricultural land and is one of the few river basins in Scotland where irrigation to produce fruit and vegetables is required. The impacts of drier summers are already being realised, as evident during the summer of 2022 when abstraction licenses for irrigation were suspended due to low flow conditions. At the same time, the basin has been impacted by flooding, likely to become a recurrent threat with projected increases in winter precipitation and extreme rainfall events [121].

The interaction between environmental conditions and human activity has led to pollution of the Eden catchment. In 2022, both the lower and upper stretches of the river were classified as in ‘moderate’ ecological status for water quality by the Scottish Environment Protection Agency (SEPA). This was attributed to discharges from wastewater treatment works, diffuse pollution from agriculture and private septic tanks. The influence of low flows on pollutant concentrations and increased source loads during high intensity rainfall events exacerbate the problem.

Tackling diffuse pollution from agriculture is a wicked problem which has not been successfully addressed through incremental changes, such as those implemented in the European Common Agricultural Policy [122] and there is increasing evidence that multiple system-based interventions will be needed to improve water quality at a catchment scale [22, 41, 42]. To this end, SEPA and Scotland’s public water supplier adopted a collaborative future-focussed systems thinking approach to decision-making in the basin.^3^ In response, Adams et al*.* (2023) co-developed a BBN as a tool for practical testing of this collaborative approach to investigate the current and future water issues. Environmental and human systems in the basin were integrated and future climatic and socio-economic scenarios were explored with stakeholders to understand the extent of future water quality and quantity issues.

The management options identified included constructed wetlands, irrigation lagoons and nutrient recycling within wastewater treatment works [48]. Compared to a linear thinking approach, the co-developed systems understanding helped to highlight ways for key stakeholders to work together across sectors, identify solutions with a potential real impact and avoid unintended consequences. Applying systems thinking approaches helped the stakeholders to move from a siloed to holistic understanding of both problems and solutions, fostering an opportunity for collaboration. However, as measures with the greatest certainty of achieving desired outcomes were also the most challenging to implement, during the feasibility assessment stage the stakeholders were still inclined to opt for easier but less certain solutions. This highlights a challenge associated with co-created system analyses. Even when following best practice in systems thinking, the insights may not be fully reflected in the resultant actions. Similar challenges have been noted across varied settings [123].

Considering these challenges, below, we highlight six aspects that should be considered to improve the integration of system analysis into decision-making and policy.

- *PLAN FOR IMPACT*: we need to start the participatory system analysis and modelling process by considering impact. What types of impact are expected and with or for whom? How do we create maximum impact from the analysis? This will determine not only the level of stakeholder participation but also the choice of modelling approach and type of model to co-construct with stakeholders. These in turn will narrow down the choice of system analysis and modelling tools. Importantly, we need to focus on achieving instrumental impact, i.e. on real and enduring transformative outcomes, and less on network-building impacts, that are a participatory means for transformation but not transformation itself.
- *DESIRABILITY and TIMELINESS:* systems thinking sometimes identifies surprises, a reality which is not always easily accommodated by policy makers. Agreeing on the research question or problem framing with policy makers will determine what is examined and what is found. However, this framing may be restrictive and will influence the desirability of the findings. Hence, it is important for researchers to get involved in the process before a question is formulated to broaden the framing and influence what outcomes may be considered ‘desirable’. Furthermore, it is essential to understand what specific institutional decision makers want in terms of decision support and at what points they need pertinent insights to be delivered to turn them into action. This requires explicit and guaranteed alignment of systems thinking processes with the decision-makers' decision-making processes.
- *SCOPE SCALABILITY:* it is important to consider the scale of interest, as responsibilities of participants will change with the scale and some phenomena will only become apparent at larger scales.
- *DEFINE SYSTEM BOUNDARIES* is critical and will be aligned with the problem framing and the scale of the enquiry. However, it is noteworthy that complex systems are permeable by definition (see [124]). Hence, any definition of a system boundary will be open to debate according to researchers, stakeholders and modellers perceptions of ‘external’ drivers as opposed to ‘internal’ dynamics in a problem domain. A key example of this is the contentious definition of a land use system in a global economy dependent on supply chains and their relation to environmental, political, and socioeconomic trade-offs [125]. Thus, whilst boundaries should be discussed and agreed specific to the problem and stakeholders, some conceptual boundary fuzziness may always be expected.
- *REVIEW MODEL CAPABILITIES:* model structure and available data will influence what we can model and there is a risk of ‘valuing what we model rather than modelling what we value’. Therefore, coupling of different approaches is key to overcoming their respective limitations to holistically represent biophysical, social and governance aspects. There is also the non-trivial problem of data needs. Having decided what needs to be modelled and how, the availability of data will influence the approach that can be taken. In practice, there is often an iteration to match data availability with the modelling approach. Hence, both model choice and available data are critical to the findings and their impact.
- *BUILD RELATIONSHIPS* with stakeholders, including policy makers and their advisors. This is critical for the ultimate credibility and salience of modelling, but it can be a difficult and time-consuming part of the process. We need to clarify how and when to incorporate multiple stakeholder values and knowledge systems, such that there is shared recognition of multiple views, which informs action, whilst also responding to the pace and scale of climate and nature crises.

These six aspects point to the need to dedicate additional resources for engagement with those involved in drafting and ultimately approving evidence-based policy, including civil servants. This requires additional skills and resources beyond those needed ‘just’ for modelling and reinforces the value of individuals and existing organisations that already act as ‘knowledge-brokers’, such as SEFARI Gateway^4^ and Centres of Expertise in Scotland.^5^ Finally, to overcome the policy-making ‘silos’, a mission-based approach to decision making may be needed, to embed cross-sectoral objectives within a systemic framework. The European Union is advocating the adoption of a long-term systems thinking approach to understand the root causes of the current crises and “Transform the economy and society through challenge-driven approaches to research and innovation” [17]. Tracking this initiative may offer useful insights for embedding systemic approaches in other contexts.

One way to foster systems thinking is to continue to communicate it, teach it, value it, and resource it in the realization of its necessity [19]. Unfortunately, before acting on this realization, we may have to experience failure due to ignorance, misunderstanding, misrecognition, or misdiagnoses of system dynamics, symptoms, and causes due to lack of joined-up approaches. Therefore, creating institutional space for systemic approaches is critical. For example, if institutions are broadly constituted of siloed departments, programmes, and actors and there is no one responsible for thinking and deciding at the system level–never mind thinking about the future–then it is hard to imagine how systems thinking might become valued or implemented. We might ask, are there many “joined-up” decision-makers? Do our cultures and institutions value such thought and activity? If not, those who seek to share systems models and tools may labour for naught.

All attempts to inform transformative change must be mindful of the limited influence which any analysis or analyst can or should expect to achieve. At least in democracies, there are many legitimate influences and inputs in policy-making. There are also many other legitimate knowledge systems to incorporate that go beyond science [126]. Therefore, we do not claim that our insights about how to target systemic analyses and processes, if used to inform other processes, will always be reflected in transformative change; however, it is important that expectations for influence are carefully articulated, planned for and used in evaluation. We propose that more transparent evaluation and reflection about effective systems approaches is needed to inform and target future systems thinking processes.

## Conclusions

Achieving sustainability goals requires more systemic approaches, but care is needed in selecting and working with tools and frameworks that are designed to achieve those objectives. The examples of systems thinking approaches in land system science that we have presented here demonstrate a variety of modelling and analytical approaches that can produce relevant insights about systems and sustainability problems; and identify some useful attributes of the analytical approaches and also – importantly – the processes of using and working with them that matter to produce insights that are useful or relevant to policymakers. However, we do not claim the approaches we have used always had a major influence on decision-makers in policy or resulted in substantial changes to policy. Some of our analyses and models have usefully helped to inform incremental or moderate change, however transformative changes as defined by the IPCC [127], i.e. those that create fundamental changes in the system, are ultimately needed to achieve sustainability [128] and must represent our ambition for the use of systems thinking approaches.

In conclusion, systems thinking approaches, whilst being essential to identify effective solutions to the complex environmental and social challenges we face, may not, by themselves, have impacts beyond informing or enabling incremental change. Therefore, we reflect on further steps that could be taken to enhance the impact of analyses and promote transformation. Approaches that are fit for purpose, identify holistic systems function, and identify the levers and leverage points are required; but also necessary is trans-disciplinary science with genuinely co-created processes. This in turn highlights the need for nuanced understanding of the drivers and constraints on decision-making, and capacity and skills to engage in evolving interactions [129]. We also need a better understanding of how effective systems thinking approaches are in driving changes in decision making. This requires not only a greater will to properly evaluate such impacts (a deficit Hedelin et al. [84] have identified), but also sustained funding for long-term evaluation of the effectiveness of systems thinking approaches based on participatory analyses, honest reflection, and open evaluation against standards. Ultimately, systems thinking approaches require supportive governance and enabling structures, to facilitate the transition from analysis to transformation.

## Data Availability

No datasets were generated or analysed during the current study.

## Abbreviations

ABM: Agent-based modelling
BBN: Bayesian belief network
CLM: Causal loop modelling
sMCA: Spatial multicriteria analysis
SMA: Societal metabolic analyses
SNM: Social Network Mapping
QST: Quantitative story telling

## Footnotes

1 Note that QST, when combined with SMA, does share the property of having a formal representation (see [85].

2 https://storymaps.arcgis.com/stories/f49964c9d7344ac4a6056cbde3122946.

3 More information on the approach, called One Planet Choices, can be found here.https://vimeo.com/802708789/e983c9867e

4 https://sefari.scot.

5 https://crew.ac.uk.

## References

[1] EGU, “The science activist: should science get Political?,” 2023. https://meetingorganizer.copernicus.org/EGU23/session/47437. Accessed 9 Feb 2024

[2] United Nations, “With world off track halfway to achieving Sustainable Development Goals, UN calls on global leaders to turbocharge action at landmark Summit,” 2023. https://www.un.org/sustainabledevelopment/blog/2023/08/media-advisory-with-world-off-track-halfway-to-achieving-sustainable-development-goals-un-calls-on-global-leaders-to-turbocharge-action-at-landmark-summit/. Accessed 9 Feb 2024

[3] EU Commission and European Commission, “The European Green Deal,” Eur. Comm 2019;53(9):24. Available: https://eur-lex.europa.eu/legal-content/EN/TXT/PDF/?uri=CELEX:52019DC0640&from=EN

[4] IPCC, “Summary for Policymakers: Synthesis Report.,” Clim. Chang. 2023 Synth. Report. Contrib. Work. Groups I, II III to Sixth Assess. Rep. Intergov. Panel Clim. Chang., pp. 1–34, 2023.

[5] Climate Change Committee, Progress in reducing emissions: 2023 Report to Parliament, no. 2023. Available: www.theccc.org.uk/publications%0Ahttps://www.theccc.org.uk/wp-content/uploads/2021/06/Progress-in-reducing-emissions-2021-Report-to-Parliament.pdf

[6] Leiserowitz M, Maibach A, Rosenthal E, Kotcher S, Ballew J, Marlon M, Carman J, Verner J, Lee M, Myers S, Goldberg T. “Global Warming’s Six Americas, December 2022.,” Yale University and George Mason University, New Haven, CT: Yale Program on Climate Change Communication, 2023. https://climatecommunication.yale.edu/publications/global-warmings-six-americas-december-2022/

[7] Raven PH, Wagner DL. Agricultural intensification and climate change are rapidly decreasing insect biodiversity. Proc Natl Acad Sci. 2021. 10.1073/pnas.2002548117. 10.1073/pnas.2002548117 PMC7812793 33431564

[8] Richardson K, et al. Earth beyond six of nine planetary boundaries. Sci Adv. 2023;9(37):1–17. 10.1126/sciadv.adh2458. 10.1126/sciadv.adh2458 PMC10499318 37703365

[9] Rockström J, et al. Safe and just earth system boundaries. Nature. 2023;619(7968):102–11. 10.1038/s41586-023-06083-8. 37258676 10.1038/s41586-023-06083-8 PMC10322705

[10] Ceballos PR, Ehrlich G. Mutilation of the tree of life via mass extinction of animal genera. Proc Natl Acad Sci. 2023;120(39):1–6. 10.1073/pnas. 10.1073/pnas.2306987120 PMC10523489 37722053

[11] Alewell C, Ringeval B, Ballabio C, Robinson DA, Panagos P, Borrelli P. Global phosphorus shortage will be aggravated by soil erosion. Nat Commun. 2020. 10.1038/s41467-020-18326-7. 10.1038/s41467-020-18326-7 PMC7486398 32917863

[12] Brownlie W, Reay DS. The Our Phosphorus Future Report 2022. 10.13140/RG.2.2.17834.08645.

[13] Homer-Dixon T, Renn O, Rockstrom J, Donges JF, Janzwood S. A call for an international research program on the risk of a global polycrisis. SSRN Electron J. 2022. 10.2139/ssrn.4058592.

[14] Stafford-Smith M, et al. Integration: the key to implementing the sustainable development goals. Sustain Sci. 2017;12(6):911–9. 10.1007/s11625-016-0383-3. 30147763 10.1007/s11625-016-0383-3 PMC6086249

[15] van den Ende MA, Hegger DLT, Mees HLP, Driessen PPJ. Wicked problems and creeping crises: a framework for analyzing governance challenges to addressing environmental land-use problems. Environ Sci Policy. 2023;141:168–77. 10.1016/j.envsci.2023.01.006.

[16] Pahl-Wostl C, Bhaduri A, Bruns A. Editorial special issue: the nexus of water, energy and food—an environmental governance perspective. Environ Sci Policy. 2018;90:161–3. 10.1016/j.envsci.2018.06.021.

[17] European Commission. Transformation in the poly- crisis age. ESIR Policy Brief. 2023. 10.2777/360282.

[18] Reynolds M, Blackmore C, Ison R, Shah R, Wedlock E. The Role of Systems Thinking in the Practice of Implementing Sustainable Development Goals. Cham: Springer International Publishing; 2018.

[19] Voulvoulis N, et al. Systems thinking as a paradigm shift for sustainability transformation. Glob Environ Chang. 2022;75: 102544. 10.1016/j.gloenvcha.2022.102544.

[20] F. McGonigle, D.F., Berry, P., Boons, “A Primer for Integrating Systems Approaches into Defra. Report from the Defra Systems Research Programme 2022. Available: https://www.gov.uk/government/publications/integrating-a-systems-approach-into-defra/integrating-a-systems-approach-into-defra#contents

[21] UKRI, “UKRI cross research council responsive mode pilot scheme: round 1,” 2023. https://www.ukri.org/opportunity/ukri-cross-research-council-responsive-mode-pilot-scheme/. Accessed 9 Feb 2024

[22] Bieroza MZ, Bol R, Glendell M. What is the deal with the Green Deal: Will the new strategy help to improve European freshwater quality beyond the water framework directive? Sci Total Environ. 2021;791: 148080. 10.1016/j.scitotenv.2021.148080. 34126496 10.1016/j.scitotenv.2021.148080

[23] Lipper L, et al. Climate-smart agriculture for food security. Nat Clim Chang. 2014;4(12):1068–72. 10.1038/nclimate2437.

[24] Wiebe K, Robinson S, Cattaneo A. Climate change, agriculture and food security. In: Sustainable Food and Agriculture. Elsevier; 2019. p. 55–74.

[25] UNFCCC. Conference of the Parties serving as the meeting of the Parties to the Paris Agreement. In: United Nations Framew. Conv. Clim. Chang. 2023. pp. 1–21

[26] Meyfroidt P, Ryan CM, Aspinall R. Clark digital commons ten facts about land systems for sustainability. Proc Natl Acad Sci. 2022;119(7):1–12. 10.1073/pnas.2109217118 PMC8851509 35131937

[27] Merriam-Webster, “System,” 2024. https://www.merriam-webster.com/dictionary/system. Accessed 9 Feb 2024

[28] Arnold RD, Wade JP. A definition of systems thinking: a systems approach. Procedia Comput Sci. 2015;44:669–78. 10.1016/j.procs.2015.03.050.

[29] Meadows D. Thinking in systems a primer. Earthscan; 2008.

[30] Schelling TC. Dynamic models of segregation†. J Math Sociol. 1971;1(2):143–86. 10.1080/0022250X.1971.9989794.

[31] Kaufman S, Boxshall A. Eleven enablers of science thought leadership to facilitate knowledge exchange in environmental regulation. Environ Sci Policy. 2023;147:336–48. 10.1016/j.envsci.2023.06.018.

[32] Reed MS, Stringer LC, Fazey I, Evely AC, Kruijsen JHJ. Five principles for the practice of knowledge exchange in environmental management. J Environ Manage. 2014;146:337–45. 10.1016/j.jenvman.2014.07.021. 25194520 10.1016/j.jenvman.2014.07.021

[33] Vertigo Ventures & Digital Science. Collecting Research Impact Evidence: Best Practice Guidance for the Research Community 2016; pp. 1–24. Available: http://www.vertigoventures.com/wp-content/uploads/2017/06/collecting-research-impact-evidence-june-2016-vv.pdf%0Ahttps://hivve.tech/resources/ebook-collecting-research-impact-evidence-2/

[34] Burrough PA, McDonnell RA, Lloyd CD. Principles of Geographical Information Systems. 3rd ed. NY: Oxford University Press; 2015.

[35] Gimona A, van der Horst D. Mapping hotspots of multiple landscape functions: a case study on farmland afforestation in Scotland. Landsc Ecol. 2007;22(8):1255–64. 10.1007/s10980-007-9105-7.

[36] Gimona A, McKeen M, Baggio A, Simonetti E, Poggio L, Pakeman RJ. Complementary effects of biodiversity and ecosystem services on spatial targeting for agri-environment payments. Land Use Policy. 2023;126: 106532. 10.1016/j.landusepol.2022.106532.

[37] Poggio L, Artz R, Gimona A. Digital soil mapping for northern peatlands: examples of mapping peats and their characteristics in Scotland, in Tropical Wetlands – innovation in mapping and management. CRC Press. 2019. 10.1201/9780429264467-4.

[38] Gagkas Z, Lilly A. Downscaling soil hydrological mapping used to predict catchment hydrological response with random forests. Geoderma. 2019;341:216–35. 10.1016/j.geoderma.2019.01.048.

[39] Poggio L, Lassauce A, Gimona A. Modelling the extent of northern peat soil and its uncertainty with Sentinel: Scotland as example of highly cloudy region. Geoderma. 2019;346:63–74. 10.1016/j.geoderma.2019.03.017.

[40] Pearl D, Mackenzie J. The Book of Why. Penguin Random House UK; 2018.

[41] Troldborg M, Gagkas Z, Vinten A, Lilly A, Glendell M. Probabilistic modelling of the inherent field-level pesticide pollution risk in a small drinking water catchment using spatial Bayesian belief networks. 2022. Hydrol Earth Syst Sci. 10.5194/hess-2021-477.

[42] Glendell M, et al. A systems approach to modelling phosphorus pollution risk in Scottish rivers using a spatial Bayesian belief network helps targeting effective mitigation measures. Front Environ Sci. 2022;10:1–22. 10.3389/fenvs.2022.976933.

[43] Gonzalez-Redin J, Luque S, Poggio L, Smith R, Gimona A. Spatial Bayesian belief networks as a planning decision tool for mapping ecosystem services trade-offs on forested landscapes. Environ Res. 2016;144:15–26. 10.1016/j.envres.2015.11.009. 26597639 10.1016/j.envres.2015.11.009

[44] Stewart GB et al. Uplandia: making better policy in complex upland systems. Final Report. 2021. Available: https://eprints.ncl.ac.uk/274791

[45] Adams KJ, et al. Developing a Bayesian network model for understanding river catchment resilience under future change scenarios. Hydrol Earth Syst Sci. 2023;27(11):2205–25. 10.5194/hess-27-2205-2023.

[46] Mentzel S, Grung M, Holten R, Tollefsen KE, Stenrød M, Moe SJ. Probabilistic risk assessment of pesticides under future agricultural and climate scenarios using a bayesian network. Front Environ Sci. 2022;10:1–17. 10.3389/fenvs.2022.957926.

[47] Martínez-Megías C, Mentzel S, Fuentes-Edfuf Y, Moe SJ, Rico A. Influence of climate change and pesticide use practices on the ecological risks of pesticides in a protected Mediterranean wetland: a Bayesian network approach. Sci Total Environ. 2023;878(2022):163018. 10.1016/j.scitotenv.2023.163018. 36963680 10.1016/j.scitotenv.2023.163018

[48] K. Adams, “Scotland’s freshwater landscape and its resilience to change: an assessment to support future policy,” University of Edinburgh, 2023. This highlights a challenge associated with co-created system analyses. Even when following best practice in ‘system thinking’ and ‘modelling’, their insights may not seem to be fully reflected in the resultant recommendations or actions. Similar challenges have been noted across varied settings (e.g. Boswell et al 2020) and related to a lack of institutional capacity for long-term planning (European Commission, 2023). An enduring challenge is how to influence governance structures to enable and.

[49] Polhill JG, et al. Crossing the chasm: a ‘tube-map’ for agent-based social simulation of policy scenarios in spatially-distributed systems. GeoInformatica. 2019;23(2):169–99. 10.1007/s10707-018-00340-z.

[50] Gonzalez-Redin J, Gordon IJ, Hill R, Polhill JG, Dawson TP. Exploring sustainable land use in forested tropical social-ecological systems: a case-study in the Wet Tropics. J Environ Manage. 2019;231:940–52. 10.1016/j.jenvman.2018.10.079. 30602255 10.1016/j.jenvman.2018.10.079

[51] Ge J, et al. Food and nutrition security under global trade: a relation-driven agent-based global trade model. R Soc Open Sci. 2021;8(1): 201587. 10.1098/rsos.201587. 33614091 10.1098/rsos.201587 PMC7890508

[52] Macal CM. Everything you need to know about agent-based modelling and simulation. J Simul. 2016;10(2):144–56. 10.1057/jos.2016.7.

[53] Janssen MA. Understanding artificial {A}nasazi,” J. Artif. Soc. Soc. Simul. 2009;12(4):13. Available: https://www.jasss.org/12/4/13.html

[54] Brown C, et al. Agent-based modeling of alternative futures in the British land use system. Earth’s Futur. 2022. 10.1029/2022EF002905.

[55] Reilly AC, Dillon RL, Guikema SD. Agent-based models as an integrating boundary object for interdisciplinary research. Risk Anal. 2021;41(7):1087–92. 10.1111/risa.13134. 29944738 10.1111/risa.13134

[56] Manson S, et al. Methodological issues of spatial agent-based models. J Artif Soc Soc Simul. 2020;23(1):3. 10.18564/jasss.4174.

[57] Farmer JD, Foley D. The economy needs agent-based modelling. Nature. 2009;460(7256):685–6. 10.1038/460685a. 19661896 10.1038/460685a

[58] Polhill G, Salt D. The importance of ontological structure: why validation by ‘Fit-to-Data’ is insufficient. Cham: Springer International Publishing; 2017. p. 141–72.

[59] Elsenbroich C, Polhill JG. Agent-based modelling as a method for prediction in complex social systems. Int J Soc Res Methodol. 2023;26(2):133–42. 10.1080/13645579.2023.2152007.

[60] Holtz G, et al. Prospects of modelling societal transitions: position paper of an emerging community. Environ Innov Soc Transit. 2015;17:41–58. 10.1016/j.eist.2015.05.006.

[61] McDowall W, Geels FW. Ten challenges for computer models in transitions research: commentary on Holtz et al. Environ Innov Soc Transit. 2017;22:41–9. 10.1016/j.eist.2016.07.001.

[62] Van Berkel DB, Verburg PH. Combining exploratory scenarios and participatory backcasting: using an agent-based model in participatory policy design for a multi-functional landscape. Landsc Ecol. 2012;27(5):641–58. 10.1007/s10980-012-9730-7. 25983392 10.1007/s10980-012-9730-7 PMC4426888

[63] Mehryar S, Sliuzas R, Schwarz N, Sharifi A, van Maarseveen M. From individual fuzzy cognitive maps to agent based models: modeling multi-factorial and multi-stakeholder decision-making for water scarcity. J Environ Manag. 2019;250: 109482. 10.1016/j.jenvman.2019.109482. 10.1016/j.jenvman.2019.109482 31494410

[64] Villamor GB, van Noordwijk M, Troitzsch KG. Triangulating agent-based models, role-playing games, and a stakeholder-centric approach to change scenarios. Curr Opin Environ Sustain. 2023;64: 101323. 10.1016/j.cosust.2023.101323.

[65] Lee J-S, et al. The complexities of agent-based modeling output analysis. J Artif Soc Soc Simul. 2015;18(4):4. 10.18564/jasss.2897.26677347

[66] Giampietro M, Mayumi K, Ramos-Martin J. Multi-scale integrated analysis of societal and ecosystem metabolism (MuSIASEM): Theoretical concepts and basic rationale. Energy. 2009;34(3):313–22. 10.1016/j.energy.2008.07.020.

[67] Windsor FM, et al. Network science: Applications for sustainable agroecosystems and food security. Perspect Ecol Conserv. 2022;20(2):79–90. 10.1016/j.pecon.2022.03.001.

[68] Timberlake TP, et al. A network approach for managing ecosystem services and improving food and nutrition security on smallholder farms. People Nat. 2022;4(2):563–75. 10.1002/pan3.10295.

[69] Tringali A, Sherer DL, Cosgrove J, Bowman R. Life history stage explains behavior in a social network before and during the early breeding season in a cooperatively breeding bird. PeerJ. 2020;8: e8302. 10.7717/peerj.8302. 32095315 10.7717/peerj.8302 PMC7020825

[70] Gao M, et al. EasyGraph: a multifunctional, cross-platform, and effective library for interdisciplinary network analysis. Patterns. 2023;4(10): 100839. 10.1016/j.patter.2023.100839. 37876903 10.1016/j.patter.2023.100839 PMC10591136

[71] Kluger LC, Scotti M, Vivar I, Wolff M. Specialization of fishers leads to greater impact of external disturbance: evidence from a social-ecological network modelling exercise for Sechura Bay, northern Peru. Ocean Coast Manag. 2019;179: 104861. 10.1016/j.ocecoaman.2019.104861.

[72] Hamilton M, Fischer AP, Ager A. A social-ecological network approach for understanding wildfire risk governance. Glob Environ Chang. 2019;54:113–23. 10.1016/j.gloenvcha.2018.11.007.

[73] Kininmonth S, Bergsten A, Bodin Ö. Closing the collaborative gap: Aligning social and ecological connectivity for better management of interconnected wetlands. Ambio. 2015;44(S1):138–48. 10.1007/s13280-014-0605-9. 10.1007/s13280-014-0605-9 PMC4289001 25576288

[74] Vignola R, McDaniels TL, Scholz RW. Governance structures for ecosystem-based adaptation: using policy-network analysis to identify key organizations for bridging information across scales and policy areas. Environ Sci Policy. 2013;31:71–84. 10.1016/j.envsci.2013.03.004.

[75] Moraine M, Duru M, Therond O. A social-ecological framework for analyzing and designing integrated crop–livestock systems from farm to territory levels. Renew Agric Food Syst. 2017;32(1):43–56. 10.1017/S1742170515000526.

[76] Collier NF, Sayer J, Boedhihartono AK, Hanspach J, Abson D, Fischer J. System properties determine food security and biodiversity outcomes at landscape scale: a case study from west flores, indonesia. Land. 2018;7(1):1–19. 10.3390/land7010039.

[77] Vennix JAM. Group Model Building. Chichester: J. Wiley; 1996.

[78] Hare M. Forms of participatory modelling and its potential for widespread adoption in the water sector. Environ Policy Gov. 2011;21(6):386–402. 10.1002/eet.590.

[79] Hare M, Pena del Valle Isla A, Perez Pena O, Toscano Alatorre AL. Upscaling Participatory Modelling for Multi-Local Community- Based Climate Change Adaptation : Methodological developments and new insights into the vulnerability of complex , socio- ecological systems Upscaling Participatory Modelling for Multi-Local Com. In: 10th International Gongress on Environmental Modelling and Software, Brussels, Belgium., London, 2020, p. 1.

[80] Hare MP, Roxburgh N, Salt D, Polhill JG. Barriers and Model Curation Issues Associated with Rapid Adaptation of Empirical Legacy ABM in Response to Urgent Policy Maker Queries. In: Proceedings of the Social Simulation Conference 2023, 4–8 September 2023, Glasgow, ESSA, 2023, pp. 1–12

[81] Hare M, Salt D, Colasanti R, Milton R, Batty M, Heppenstall A, Polhill G. Taking Agent-Based Social Simulation to the Next Level Using Exascale Computing: potential use cases, capabilities and threats. In: Proceedings of the 23rd International Conference on Autonomous Agents and Mult-Agent Systems (AAMAS 2024) May 6–10 Auckland, New Zealand., IFAAMAS.

[82] Pena Del Valle Isla A, Hare M, Perez Pena O. Participatory systems modelling to explore the systemic drivers of the deterioration of food system resilience at different spatial scales: A study of rapid transition from rural to periurban livelihoods and food insecurity, Session on” Building and conce. In: Royal Geographical Society-IBG Conference, 29 August - 1 September 2023, London., 2023.

[83] Kuipers BJ. Qualitative simulation: then and now. Artif Intell. 1993;59(1–2):133–40. 10.1016/0004-3702(93)90179-F.

[84] Hedelin B, et al. What’s left before participatory modeling can fully support real-world environmental planning processes: a case study review. Environ Model Softw. 2021;143: 105073. 10.1016/j.envsoft.2021.105073.

[85] Matthews KB, et al. Old wine in new bottles: exploiting data from the EU’s farm accountancy data network for pan-EU sustainability assessments of agricultural production systems. Sustainability. 2021;13(18):10080. 10.3390/su131810080.

[86] Giampietro M, Aspinall RJ, Ramos-Martin J, Bukkens SGF, editors. Resource Accounting for Sustainability Assessment. Routledge; 2014. 10.4324/9781315866895.

[87] Jakeman AJ, Letcher RA, Norton JP. Ten iterative steps in development and evaluation of environmental models. Environ Model Softw. 2006;21(5):602–14. 10.1016/j.envsoft.2006.01.004.

[88] Cash D, Clark WC, Alcock F, Dickson N, Eckley N, Jger J. Salience, credibility, legitimacy and boundaries: linking research, assessment and decision making. SSRN Electron J. 2003. 10.2139/ssrn.372280.

[89] Matthews KB, Buchan K, Miller DG, Towers W. Reforming the CAP—with area-based payments, who wins and who loses? Land Use Policy. 2013;31:209–22. 10.1016/j.landusepol.2012.06.013.

[90] Cattaneo C, Marull J, Tello E. Landscape agroecology. The dysfunctionalities of industrial agriculture and the loss of the circular bioeconomy in the Barcelona Region. Sustain. 2018. 10.3390/su10124722.

[91] Polhill JG, Gimona A, Gotts NM. Nonlinearities in biodiversity incentive schemes: a study using an integrated agent-based and metacommunity model. Environ Model Softw. 2013;45:74–91. 10.1016/j.envsoft.2012.11.011.

[92] Adams K et al. 2024 Identifying and testing adaptive management options to increase river catchment system resilience using a Bayesian Network model 2024: pp. 1–24. Available: 10.21203/rs.3.rs-4172006/v1

[93] Elsenbroich C. Explanation in agent-based modelling: functions, causality or mechanisms. JASS. 2012. 10.18564/jasss.1958.

[94] Lansing JN, Kremer JS. Emergent properties of balinese water temple networks: coadaptation on a rugged fitness landscape. Am Anthropol. 1993. 10.1525/aa.1993.95.1.02a00050.

[95] Negri C, et al. Transferability of a Bayesian belief network across diverse agricultural catchments using high-frequency hydrochemistry and land management data. Sci Total Environ. 2024. 10.1016/j.scitotenv.2024.174926. 10.1016/j.scitotenv.2024.174926 39059662

[96] Uribe Camacho A León Corrales MA López Gijón G, Peña del Valle Isla A, Hare M.P, Torres GUribe Camacho A, León Corrales M.A, López Gijón G, Peña del Valle Isla A, Hare M.P, Torres González L.G, Davydova Belitskaya V, Garza Galic. PACMUBIS – Programa de Acción Climática Municipal para el Bienestar y la Sustentabilidad para el Municipio de Tlajomulco de Zúñiga, Jalisco (PACMUBIS – Municipal Programme on Climate Action for Wellbeing and Sustainability for the Municipality of Tlajomul 2018.

[97] Daniell KA, White I, Ferrand N, Ribarova IS, Coad P, Rougier J-E, Hare M, Jones NA, Popova A, Rollin D, Perez P, Burn S. Co-engineering participatory water management processes: theory and insights from Australian and Bulgarian interventions. Ecol Soc 2010;15(4). Available: http://www.ecologyandsociety.org/vol15/iss4/art11/

[98] Guerrero AM, Bodin Ö, McAllister RRJ, Wilson KA. Achieving social-ecological fit through bottom-up collaborative governance: an empirical investigation. Ecol Soc. 2015. 10.5751/ES-08035-200441.

[99] Matthewsa KB, Blackstocka KL, Wardell-Johnsona DH, Millera DG, Tavanaa M, Thomsonb S, Moxeyc A, Neilsona R, Baggaleya N, Gilesa M, Karleya A. Synthesis Report: Screening Enhanced Conditionality Measures. An output of RESAS commissioned project Supporting Scotland’s Land Use Transformations (JHI-C3–1) 2023. Available: https://landusetransformations.hutton.ac.uk/outputs/d4-enhanced-conditionality-screening-synthesis

[100] Mzyece CC, et al. Eliciting expert judgements to underpin our understanding of faecal indicator organism loss from septic tank systems. Sci Total Environ. 2024. 10.1016/j.scitotenv.2024.171074. 10.1016/j.scitotenv.2024.171074 38378059

[101] Barreteau O. Our Companion Modelling Approach. J Artif Soc. Soc Simul 2003;6. Available: https://www.jasss.org/6/2/1.html

[102] Basco-Carrera L, et al. An adapted companion modelling approach for enhancing multi-stakeholder cooperation in complex river basins. Int J Sustain Dev World Ecol. 2018;25(8):747–64. 10.1080/13504509.2018.1445668.

[103] Simon C, Etienne M. A companion modelling approach applied to forest management planning. Environ Model Softw. 2010;25(11):1371–84. 10.1016/j.envsoft.2009.09.004.

[104] Le Page C, Naivinit W, Trébuil G, Gajaseni N. Companion Modelling with Rice Farmers to Characterise and Parameterise an Agent-Based Model on the Land/Water Use and Labour Migration in Northeast Thailand. In: Smajgl A, Barreteau O, editors. Empirical agent-based modelling - challenges and solutions. New York: Springer; 2014. p. 207–21.

[105] Pahl-Wostl C, Hare M. Processes of social learning in integrated resources management. J Community Appl Soc Psychol. 2004;14(3):193–206. 10.1002/casp.774.

[106] Giordano R, et al. Combining social network analysis and agent-based model for enabling nature-based solution implementation: the case of Medina del Campo (Spain). Sci Total Environ. 2021;801: 149734. 10.1016/j.scitotenv.2021.149734. 34467897 10.1016/j.scitotenv.2021.149734

[107] Linda May LG, Glendell M, Adams K, Gagkas Z, Iain Gunn EZ, Hannah M, Roberts M, Spears B, Taylor P, Thackeray S, Troldborg M. Mitigating Climate Change Impacts on the Water Quality of Scottish Standing Waters. 2024. Available: https://www.crew.ac.uk/publication/assessing-climate-change-impacts-water-quality-scottish-standing-waters

[108] Van Voorn G, Hengeveld G, Verhagen J. An agent based model representation to assess resilience and efficiency of food supply chains. PLoS ONE. 2020;15(11): e0242323. 10.1371/journal.pone.0242323. 33211734 10.1371/journal.pone.0242323 PMC7676680

[109] Lam DPM, Martín-López B, Horcea-Milcu AI, Lang DJ. A leverage points perspective on social networks to understand sustainability transformations: evidence from Southern Transylvania. Sustain Sci. 2021;16(3):809–26. 10.1007/s11625-020-00881-z.

[110] Mentzel S, et al. Using a Bayesian network model to predict risk of pesticides on aquatic community endpoints in a rice field—a southern european case study. Environ Toxicol Chem. 2023. 10.1002/etc.5755. 10.1002/etc.5755 37750580

[111] Wilkinson MD, et al. The FAIR guiding principles for scientific data management and stewardship. Sci Data. 2016;3(1): 160018. 10.1038/sdata.2016.18. 26978244 10.1038/sdata.2016.18 PMC4792175

[112] Moallemi EA, et al. A review of systems modelling for local sustainability. Environ Res Lett. 2021. 10.1088/1748-9326/ac2f62.

[113] Borowski I, Hare M. Exploring the gap between water managers and researchers: difficulties of model-based tools to support practical water management. Water Resour Manag. 2007;21(7):1049–74. 10.1007/s11269-006-9098-z.

[114] Young JC, et al. Improving the science-policy dialogue to meet the challenges of biodiversity conservation: having conversations rather than talking at one-another. Biodivers Conserv. 2014;23(2):387–404. 10.1007/s10531-013-0607-0.

[115] Bergmann M, et al. Transdisciplinary sustainability research in real-world labs: success factors and methods for change. Sustain Sci. 2021;16(2):541–64. 10.1007/s11625-020-00886-8.

[116] Norström AV, et al. Principles for knowledge co-production in sustainability research. Nat Sustain. 2020;3(3):182–90. 10.1038/s41893-019-0448-2.

[117] Chilvers J, Kearnes M, editors. Remaking Participation. NY: Routledge; 2015. 10.4324/9780203797693.

[118] Cash DW, et al. Knowledge systems for sustainable development. Proc Natl Acad Sci. 2003;100(14):8086–91. 10.1073/pnas.1231332100. 12777623 10.1073/pnas.1231332100 PMC166186

[119] Bodin Ö. “Collaborative environmental governance: Achieving collective action in social-ecological systems. Science. 2017. 10.1126/science.aan1114. 10.1126/science.aan1114 28818915

[120] Rockström J, et al. The unfolding water drama in the Anthropocene: towards a resilience-based perspective on water for global sustainability. Ecohydrology. 2014;7(5):1249–61. 10.1002/eco.1562.

[121] Werritty A, Sugden D. Climate change and Scotland: recent trends and impacts. Earth Environ Sci Trans R Soc Edinburgh. 2012;103(2):133–47. 10.1017/S1755691013000030.

[122] EEA, “Rethinking agriculture. Briefing No. 25/2021,” 2021. https://www.eea.europa.eu/publications/rethinking-agriculture. Accessed 9 Feb 2024

[123] Boswell J, Baird J, Taheem R. The challenges of putting systems thinking into practice comment on "what can policy-makers get out of systems thinking? policy partners’ experiences of a systems-focused research collaboration in preventive health". Int J Heal Policy Manag. 2020. 10.34172/ijhpm.2020.92. 10.34172/ijhpm.2020.92 PMC9056187 32610827

[124] Thurner S, Klimek P, Hanel R. Introduction to the Theory of Complex Systems, vol. 1. Oxford University Press; 2018. 10.1093/oso/9780198821939.001.0001.

[125] Friis C, Nielsen J. On the system. Boundary choices, implications, and solutions in telecoupling land use change research. Sustainability. 2017. 10.3390/su9060974.

[126] Díaz S, et al. The IPBES conceptual framework—connecting nature and people. Curr Opin Environ Sustain. 2015;14:1–16. 10.1016/j.cosust.2014.11.002.

[127] IPCC. Annex I: glossary in global warming of 15°C. Cambridge University Press; 2022. p. 541–62. 10.1017/9781009157940.008.

[128] Sachs JD, Schmidt-Traub G, Mazzucato M, Messner D, Nakicenovic N, Rockström J. Six transformations to achieve the sustainable development goals. Nat Sustain. 2019;2(9):805–14. 10.1038/s41893-019-0352-9.

[129] Waylen KA, et al. Post-normal science in practice: Reflections from scientific experts working on the European agri-food policy nexus. Environ Sci Policy. 2023;141:158–67. 10.1016/j.envsci.2023.01.007.

[130] Chattoe-Brown E. Is agent-based modelling the future of prediction? Int J Soc Res Methodol. 2023;26(2):143–55. 10.1080/13645579.2022.2137923.

[131] Edmonds B. The practice and rhetoric of prediction – the case in agent-based modelling. Int J Soc Res Methodol. 2023;26(2):157–70. 10.1080/13645579.2022.2137921.

